# Schisanhenol Alleviates Mycophenolic Acid-Induced Intestinal Epithelial Cell Barrier Damage by Activating the Nrf2/HO-1 Signaling Pathway

**DOI:** 10.5812/ijpr-161994

**Published:** 2025-07-26

**Authors:** Yiyun Deng, Jiawang Fan, Chunlan Yang, Yan Huang, Qingrong Xia, Jun Liang, Dujuan Xu, Yan Du, Yong Su

**Affiliations:** 1 Department of Pharmacy, Affiliated Psychological Hospital of Anhui Medical University, Hefei, China; 2 Department of Pharmacy, Hefei Fourth People's Hospital, Hefei, China; 3 School of Pharmacy, Anhui Medical University, Hefei, China; 4 Department of Pharmacy, First Affiliated Hospital of Anhui Medical University, Hefei, China

**Keywords:** Schisanhenol, Mycophenolic Acid, Tight Junctions, Reactive Oxygen Species, Antioxidant

## Abstract

**Background:**

Reactive oxygen species (ROS)-mediated apoptosis of intestinal epithelial cells and tight junction (TJ) protein loss play critical roles in mycophenolic acid (MPA)-induced disruption of intestinal epithelial barrier function, yet no effective therapeutic strategies exist. Schisanhenol (Sal), a major component of the traditional Chinese medicine Wuzhi capsule, exhibits strong antioxidant activity.

**Objectives:**

The present study aimed to investigate the protective efficacy and mechanisms of Sal against MPA-induced damage to the intestinal mechanical barrier.

**Methods:**

Caco-2 cells were exposed to MPA (10 μM), Sal (5, 10, and 25 μM), or ML385 (2 μM) for 24 hours. Cell viability was measured via a Cell Counting Kit-8 (CCK-8) assay. The expression of apoptosis-related and TJ proteins was evaluated through Western blot analysis. Flow cytometry was used to quantify the percentage of apoptotic Caco-2 cells. Immunofluorescence assays were performed to assess the localization of TJ proteins. Intracellular ROS levels were measured using H2DCFDA staining. Oxidative and antioxidative biomarker levels were quantified using specific assay kits.

**Results:**

Sal significantly increased the viability of MPA-treated cells. It also upregulated Bcl-2 expression and reduced apoptosis. Furthermore, Sal increased the expression of the TJ proteins ZO-1 and occludin. Additionally, Sal upregulated the MPA-mediated decrease in Nrf2/HO-1 expression, reduced intracellular ROS accumulation, and increased the levels of antioxidants, including SOD, CAT, and GSH. However, ML385 partially abrogated the protective effects of Sal.

**Conclusions:**

Schisanhenol alleviates MPA-induced intestinal mechanical barrier damage via modulation of the Nrf2 signaling pathway, highlighting its antioxidant and antiapoptotic properties.

## 1. Background

Mycophenolic acid (MPA) is a widely used immunosuppressive agent in kidney transplant patients. However, approximately 50% of patients suffer from severe gastrointestinal symptoms after MPA administration, with watery, nonfebrile diarrhea being the most prevalent manifestation (1). Currently, the only available strategies are dose reduction or discontinuation of MPA, which substantially increase the risk of graft rejection (2). Therefore, identifying effective treatments to prevent MPA-induced diarrhea is essential for improving long-term graft outcomes.

The intestinal mechanical barrier, which is composed of intestinal epithelial cells and intercellular tight junctions (TJs), plays a vital role in preserving intestinal homeostasis (3). Disruption of TJs impairs the mucosal barrier, increases intestinal permeability, and thereby allows bacteria and other harmful substances to migrate into the intestinal lumen, triggering inflammatory responses and ultimately contributing to the development of intestinal disorders (4, 5). The TJs, which are located at the apical region of epithelial cells, are composed of various proteins, including transmembrane proteins such as claudin and occludin and the cytoplasmic scaffolding protein ZO-1. The ZO family maintains epithelial integrity and promotes cell proliferation (6), while the C-terminal domain of occludin interacts with ZO-1, which is pivotal in regulating TJ assembly (7). Our previous studies demonstrated that reactive oxygen species (ROS)-mediated apoptosis of intestinal epithelial cells, along with the downregulation of TJ proteins such as occludin and ZO-1, are major contributors to MPA-induced disruption of the intestinal barrier, ultimately contributing to diarrhea (8). Therefore, antioxidant-based pharmacological agents are considered promising candidates for alleviating MPA-induced intestinal barrier damage. Nuclear factor erythroid 2-related factor 2 (Nrf2) is a key transcription factor that governs the cellular redox balance. Activation of the Nrf2/heme oxygenase-1 (HO-1) signaling pathway helps preserve TJ architecture and increases intestinal barrier integrity (9).

*Schisandra chinensis*, a traditional Chinese medicinal herb with a long history of use, is recognized for its hepatoprotective, nephroprotective, antidiarrhoeal, and antimicrobial properties (10). Clinically, our team reported that Wuzhi capsule (WZ) derived from the ethanol extract of *S. chinensis* effectively alleviate MPA-induced diarrhea in renal transplant recipients. Schisanhenol (Sal), a major antioxidant lignin isolated from *S. chinensis*, has shown therapeutic potential in neurological and cardiovascular disorders by enhancing tissue antioxidant defenses and protecting against oxidative stress induced by ROS (11-14). However, the effects of Sal on intestinal disorders remain underexplored, and its impact on MPA-induced intestinal mechanical barrier injury has not been clearly elucidated.

## 2. Objectives

The present study aimed to investigate whether Sal protects against MPA-induced Caco-2 cell damage through the activation of the Nrf2/HO-1 antioxidant system.

## 3. Methods

### 3.1. Chemicals and Reagents

Schisanhenol (purity ≥ 98%, CAS: 69363-14-0) and MPA (purity ≥ 98%, CAS: 24280-93-1) were purchased from Yuanye Biological Technology Co., Ltd. (Shanghai, China). ML385 (HY-100523) was purchased from MedChemExpress (Shanghai, China). The following specific antibodies were purchased from different companies: Anti-GAPDH (1:10000; Abcam, UK); anti-Bcl-2, anti-Bax (1:1000; Abcam, UK); anti-ZO-1, anti-Nrf2, anti-HO-1 (1:1000; Affinity Bioscience, China); and anti-occludin (1:1000; ZENBio, China) antibodies.

### 3.2. Cell Culture

Human colon adenocarcinoma Caco-2 cells (Wuhan Punosai Life Science and Technology Co., Ltd.) were used as an in vitro model of intestinal epithelial cells. The cells were resuscitated and cultured in DMEM supplemented with 10% fetal bovine serum (FBS) at 37 °C in a humidified incubator with 5% CO_2_. The cells were maintained until they reached confluence and then differentiated into enterocyte-like cells.

### 3.3. Cell Viability Assay

Caco-2 cells were digested with trypsin and seeded in 96-well plates at a density of ~4,000 cells per well. Upon reaching ~70% confluence, the cells were treated with varying concentrations of MPA (10, 20, 40, 80, or 100 μM), Sal (2.5, 5, 10, 25, 50, or 75 μM), or combinations of MPA (10 μM) with Sal (2.5 - 50 μM) for 24 hours. Subsequently, CCK-8 reagent was added and incubated for 1 hour. The absorbance was measured at 450 nm using a microplate reader (BIOTEK, Vermont, USA). Cell viability was calculated as follows:

Viability (%) = (OD of treatment group/OD of control group) × 100%

### 3.4. Western Blotting

The cells were lysed in RIPA buffer (with protease inhibitors) on ice for 30 minutes. The lysates were centrifuged at 12000 rpm for 20 minutes at 4°C, and the supernatants were mixed with loading buffer at a 1:4 ratio and boiled for 10 minutes. Proteins were separated using SDS-PAGE and transferred to PVDF membranes under a constant current (220 mA, 80 minutes) in an ice bath. The membranes were blocked and incubated overnight at 4°C with primary antibodies. After washing, the membranes were incubated with HRP-conjugated secondary antibodies for 1 hour at 37°C, and signals were visualized using a chemiluminescent substrate.

### 3.5. Immunofluorescence

Cells were seeded in 24-well plates and, after treatment, fixed with 4% paraformaldehyde for 15 minutes and rinsed with PBS. Nonspecific binding was blocked with 5% BSA for 30 minutes. The cells were incubated overnight at 4°C with primary antibodies against ZO-1 (1:200, Affinity Biosciences) and occludin (1:250, ZENBio). The following day, fluorescently labelled secondary antibodies were applied for 1 hour, followed by DAPI staining (10 minutes, room temperature). Images were captured using a fluorescence inverted microscope.

### 3.6. Apoptosis Analysis

Caco-2 cells were seeded in 6-well plates and treated with MPA and/or Sal for 24 hours. After digestion with EDTA-free trypsin, the cells were collected by centrifugation at 1500 rpm for 5 minutes and washed twice with PBS. Annexin V binding buffer (400 μL) and annexin V-FITC (4 μL) were added to each sample and incubated in the dark for 15 minutes, followed by the addition of 8 μL of PI staining solution. The samples were analyzed immediately by flow cytometry. Flow cytometry gating strategy are provided in Appendix 1 in the Supplementary File.

### 3.7. Intracellular Reactive Oxygen Species Level Detection

The cells were cultured in 6-well or 24-well plates and treated with MPA and/or Sal for 24 hours. The H2DCFDA stock was diluted with PBS to obtain a 5 μM working solution. After digestion and collection, the cells were incubated with H2DCFDA (1:1 ratio with suspension) at 37°C for 20 minutes in the dark. The mean fluorescence intensity was then assessed using a flow cytometer (Beckman Coulter, USA) or an inverted fluorescence microscope (Zeiss, Germany). Flow cytometry gating strategy is provided in Appendix 2 in the Supplementary File.

### 3.8. Antioxidant Index Measurements

The cells were cultured in 10 cm dishes. After treatment, the cells were washed twice with PBS and lysed in RIPA buffer containing PMSF for 30 minutes. The lysates were centrifuged at 1000 rpm for 10 minutes, and the protein concentration in the supernatant was determined using a BCA kit. In accordance with each kit’s protocol, the levels of MDA (530 nm), SOD (450 nm), CAT (510 nm), GSH (405 nm), and GSSG (412 nm) were measured using a microplate reader and calculated via standard formulas.

### 3.9. Statistical Analysis

The data are expressed as the means ± standard deviations (SDs). Statistical analysis was conducted using SPSS 20.0 software, and graphical plotting was performed with GraphPad Prism. All the experiments were conducted in triplicate. One-way ANOVA followed by Tukey’s post hoc test was used to compare differences between groups. A P-value < 0.05 was considered statistically significant.

## 4. Results

### 4.1. Effects of Schisanhenol and Mycophenolic Acid on the Viability of Caco-2 Cells

**Figure 1.**
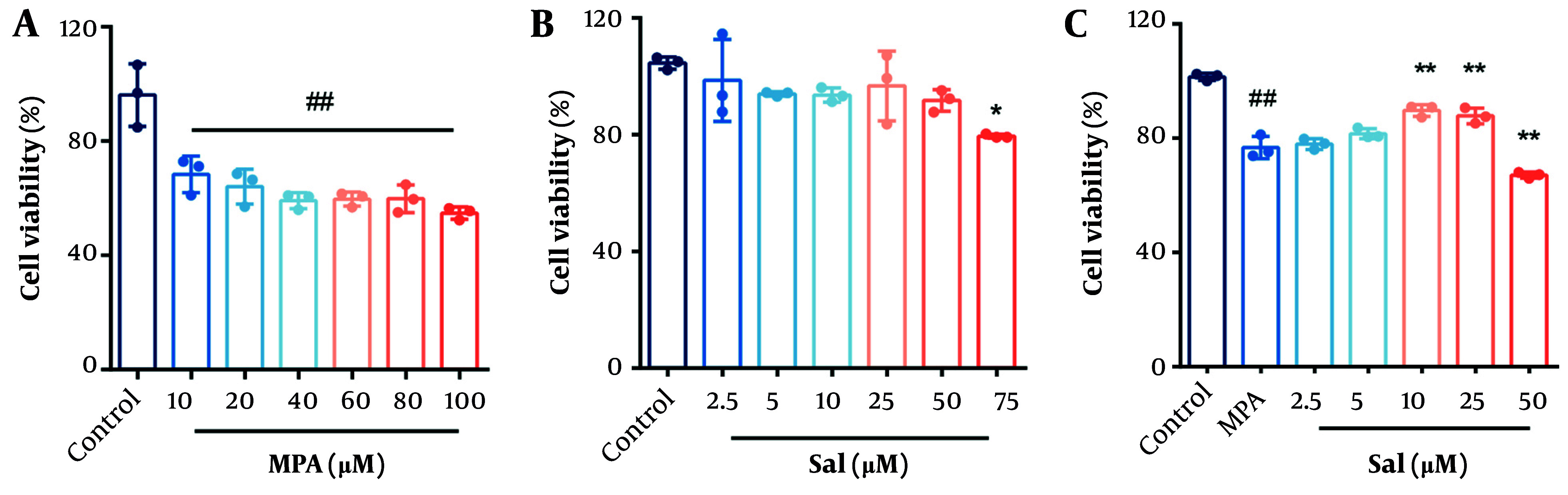
A and B, effects of Schisanhenol (Sal) and mycophenolic acid (MPA) on the viability of Caco-2 cells. Caco-2 cells were treated with different concentrations of MPA (10 - 100 μM) and Sal (2.5 - 75 μM) alone for 24 hours; C, 10 μM MPA was co-treated with different concentrations of Sal (2.5 - 50 μM) for 24 hours. Every group n = 3. * P < 0.05 and ** P < 0.01 significantly different from the MPA group. #, ## P < 0.05 and 0.01 were significantly different compared to the control group. The data are shown as the mean ± SD of n = 3.

Caco-2 cells were treated with varying concentrations of MPA and Sal for 24 hours, and cell viability was assessed using the CCK-8 assay to determine the optimal concentrations for subsequent experiments. As shown in Figure 1, cell viability significantly decreased in the MPA group starting at a concentration of 10 μM compared with the control group (Figure 1A). Schisanhenol inhibited cell viability starting at a concentration of 75 μM (Figure 1B). To evaluate the effect of Sal on Caco-2 cell viability following MPA treatment, 10 μM MPA was selected for further experiments. Cotreatment of Caco-2 cells with MPA and 2.5 - 50 μM Sal significantly ameliorated the MPA-induced reduction in cell viability within the 5 - 25 μM range (Figure 1C). 

### 4.2. Inhibition of Mycophenolic Acid-Induced Apoptosis in Caco-2 Cells by Schisanhenol

**Figure 2.**
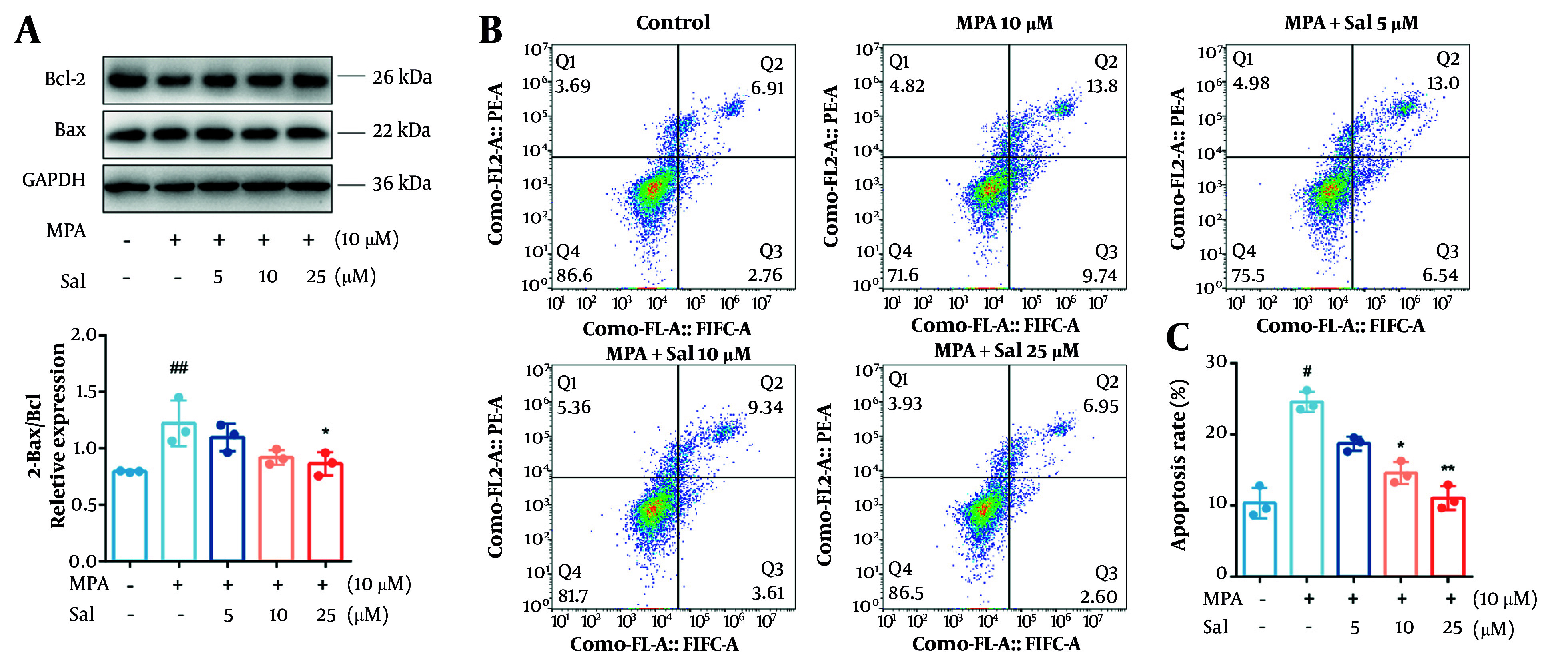
Schisanhenol (Sal) reduced apoptosis in Caco-2 cells caused by mycophenolic acid (MPA). Cells were treated with 10 μM MPA and different concentrations of Sal (5, 10, 25 μM) for 24 hours. A, apoptosis-related protein levels were detected by Western blot; and B and C, apoptosis was determined by flow cytometry. *, ** P < 0.05 and 0.01 were significantly different from the MPA group. # P < 0.05 and ## P < 0.01 significantly different compared to control group. The data are shown as the mean ± SD of n = 3.

To investigate the effect of Sal on apoptosis, we first performed a Western blot analysis to measure the expression levels of the antiapoptotic protein Bcl-2 and the proapoptotic protein Bax. The results indicated that MPA treatment did not significantly affect Bax expression; however, it significantly reduced Bcl-2 expression and decreased the Bcl-2/Bax ratio. In contrast, co-treatment with MPA and Sal resulted in a dose-dependent increase in Bcl-2 expression and an elevated Bcl-2/Bax ratio (Figure 2A). Furthermore, Sal significantly reduced the proportion of apoptotic cells (Figure 2B and C).

### 4.3. Schisanhenol’s Inhibition of Mycophenolic Acid-Induced Disruption of Tight Junction Structure in Caco-2 Cells

**Figure 3.**
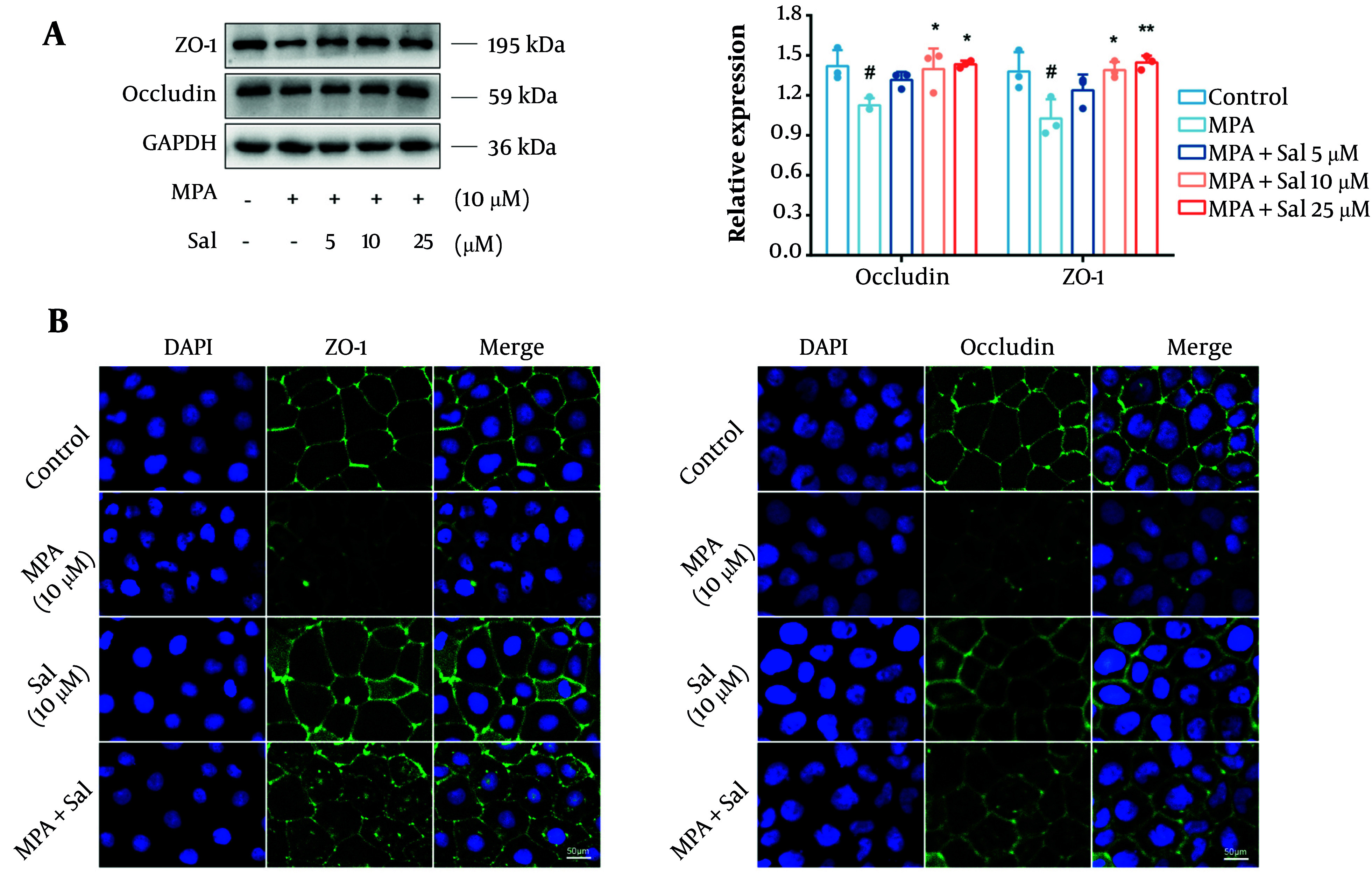
Schisanhenol (Sal) inhibited mycophenolic acid (MPA) to disrupt the tight junction (TJ) structure of Caco-2 cells. 10 μM MPA and 5, 10, 25 μM Sal were treated with Caco-2 cells for 24 hours. A and B, Western blot and immunofluorescence experiments were performed to determine the expression and distribution of occludin and ZO-1. DAPI showed blue fluorescence, occludin and ZO-1 showed green fluorescence. *, ** P < 0.05 and 0.01 were significantly different from the MPA group. # P < 0.05 significantly different from the control group. The data are shown as the mean ± SD of n = 3.

We initially assessed the effects of Sal on the expression levels of the TJ proteins occludin and ZO-1 using a Western blot analysis. The results demonstrated a significant reduction in the expression of occludin and ZO-1 in the MPA group compared with the control group. However, cotreatment with Sal resulted in a dose-dependent increase in the expression of these proteins (Figure 3A). Additionally, immunofluorescence analysis revealed that Sal effectively restored the MPA-damaged TJ structure (Figure 3B). 

### 4.4. Inhibition of Mycophenolic Acid-Induced Reactive Oxygen Species Overaccumulation by Schisanhenol

**Figure 4.**
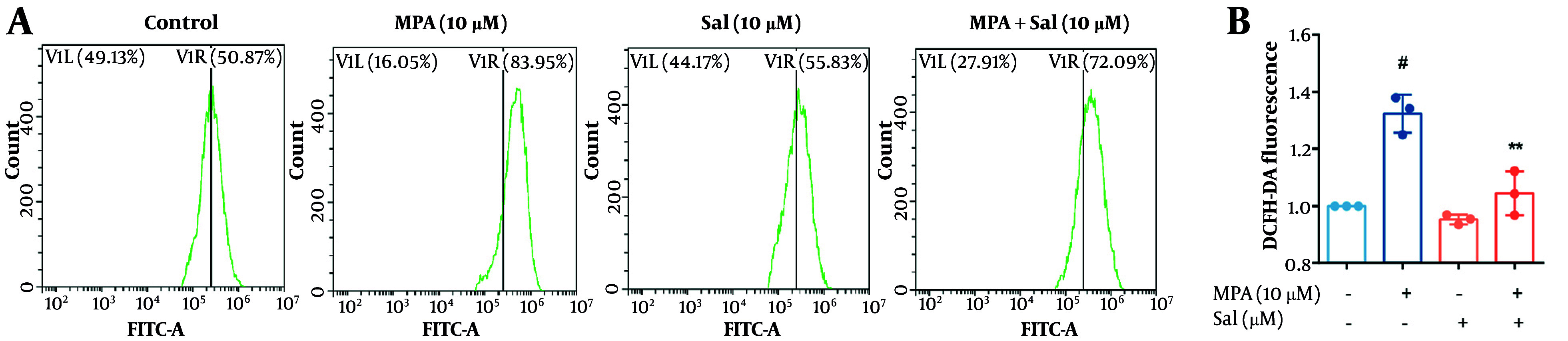
Schisanhenol (Sal) inhibited mycophenolic acid (MPA)-induced reactive oxygen species (ROS) overaccumulation. After 24 hours of treatment with 10 μM Sal and 10 μM MPA, A, the H2DCFDA probe was loaded and the average fluorescence intensity was detected by flow cytometry to assess intracellular ROS levels. B, quantification of A. ** P < 0.01 significantly different from MPA group. # P < 0.05 significantly different compared to the control group. The data are shown as the mean ± SD of n = 3.

We evaluated the effect of Sal on ROS generation using the H2DCFDA fluorescent probe. As depicted in Figure 4, MPA treatment alone resulted in a significant increase in ROS levels compared with those in the control group. In contrast, Sal treatment alone did not significantly affect ROS generation. However, co-treatment with Sal and MPA significantly reduced ROS levels. These findings suggest that MPA induces an abnormal accumulation of ROS in intestinal epithelial cells. Conversely, Sal effectively scavenges the elevated ROS induced by MPA, thereby preserving the homeostasis of intestinal epithelial cells.

### 4.5. Modulation of the Nrf2/HO-1 Pathway by Schisanhenol to Enhance Antioxidant Capacity in Caco-2 Cells

**Figure 5.**
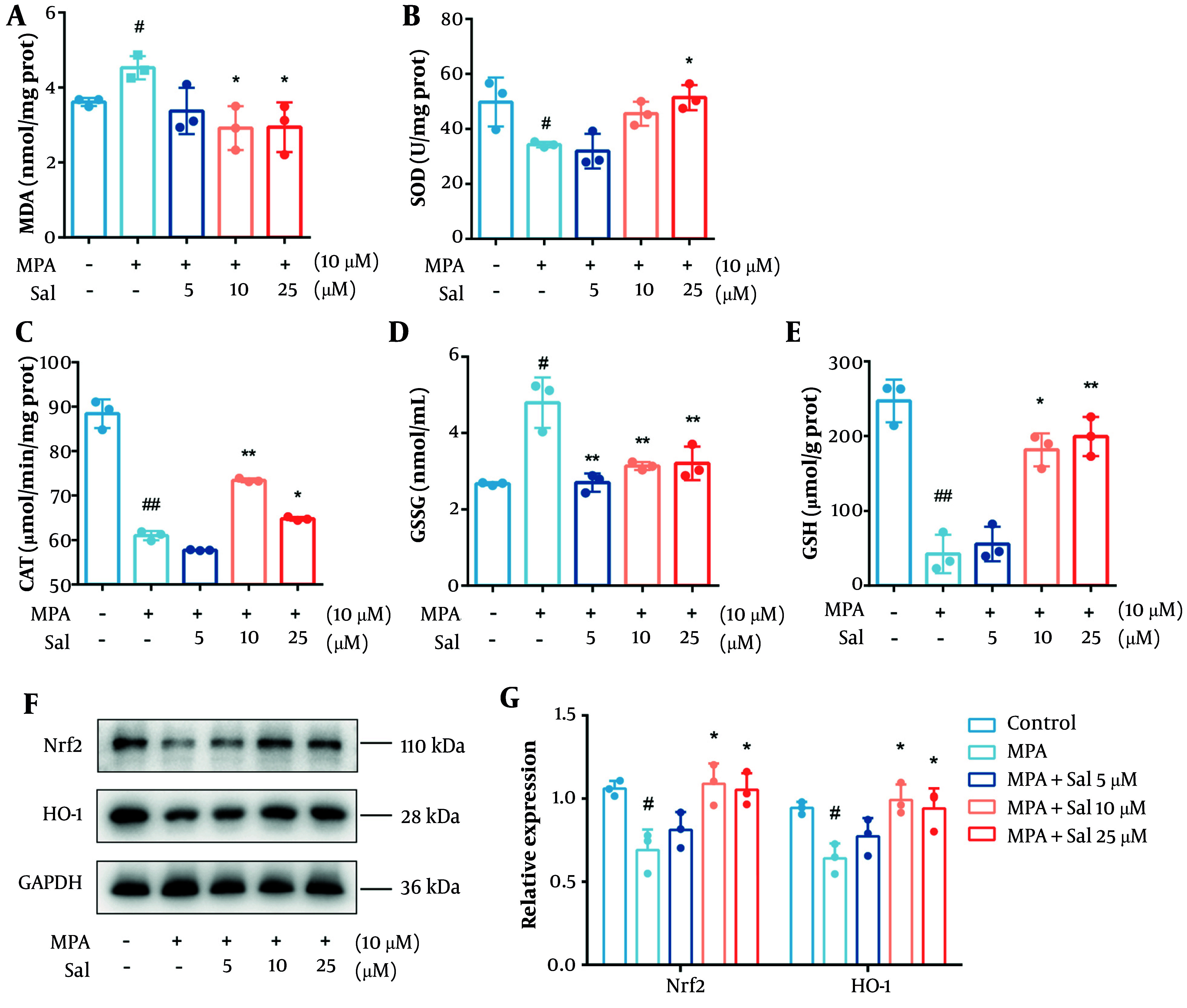
Schisanhenol (Sal) modulated the Nrf2/HO-1 pathway to improve antioxidant capacity in Caco-2 cells. After 24 hours of treatment with Sal (5, 10, 25 μM) and 10 μM mycophenolic acid (MPA), the kit detected A, MDA; B, SOD; C, CAT; D, GSSG; E, GSH content; and F and G, western blot determined Nrf2, HO-1 protein expression levels. * P < 0.05, ** P < 0.01 Significantly different from MPA group. # P < 0.05, ## P < 0.01 significantly different from control group. The data are shown as the mean ± SD of n = 3.

It is well established that glutathione predominantly exists in its reduced form but is converted to its oxidized form under oxidative stress. The depletion of antioxidant enzymes and accumulation of MDA are also indicators of oxidative stress. Therefore, we first evaluated the effects of Sal on the MDA, SOD, CAT, GSH, and GSSG levels. As expected, Sal treatment decreased the GSH and MDA levels while increasing the intracellular levels of SOD, CAT, and GSSG (Figure 5A-E). We then examined the protein expression of Nrf2 and HO-1 in Caco-2 cells. The results showed a significant increase in the expression of Nrf2 and HO-1 in the Sal group, suggesting that Sal activated the Nrf2/HO-1 pathway previously inhibited by MPA (Figure 5F-G). 

### 4.6. Abolishment of Schisanhenol’s Antioxidant and Anti-Barrier Damage Effects Through Nrf2 Inhibition

**Figure 6.**
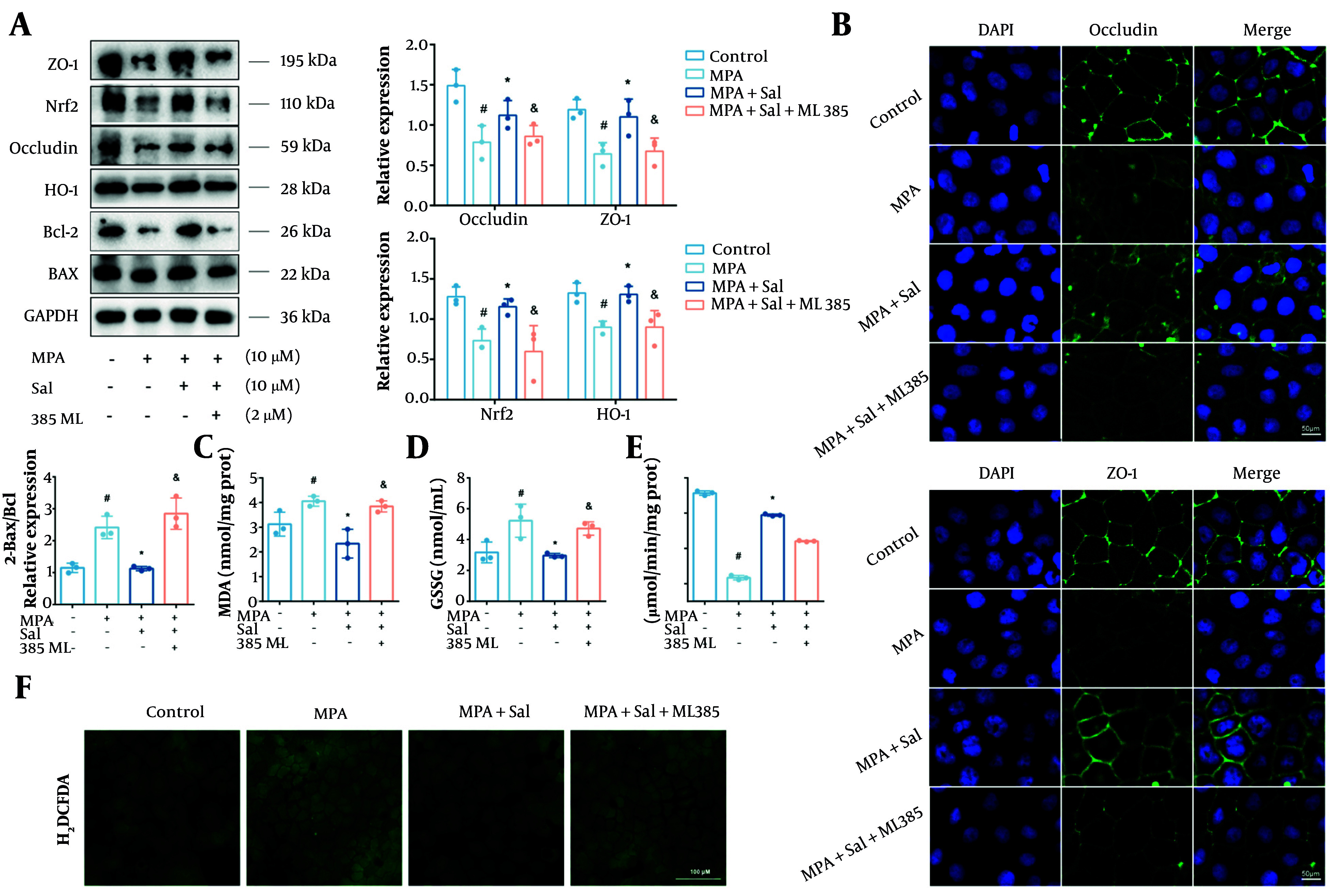
Inhibition of Nrf2 abolished the antioxidant and anti-barrier damage effects of Schisanhenol (Sal). A, after 24 hours of treatment with Sal (5, 10, 25 μM) and 10 μM mycophenolic acid (MPA), Bax, Bcl-2, occludin, ZO-1, Nrf2 and HO-1 protein levels were detected by Western blot; B, immunofluorescence experiments were performed to determine the expression and distribution of occludin and ZO-1. DAPI showed blue fluorescence, occludin and ZO-1 showed green fluorescence; The kit detected C, MDA; D, GSSG; E, CAT; F, the H2DCFDA probe was loaded and detected by fluorescence inverted microscope to assess intracellular reactive oxygen species (ROS) levels. * P < 0.05 significantly different from MPA group. # P < 0.05 significantly different from control group. & P < 0.05 significantly different from MPA + Sal group. The data are shown as the mean ± SD of n = 3.

To further investigate whether Nrf2 is a critical target of Sal, Caco-2 cells were treated with the Nrf2-specific inhibitor ML385. The results show that, cotreatment with ML385 markedly abrogated protective effects of Sal, as evidenced by decreased expression of occludin, ZO-1, and Bcl-2, along with elevated ROS levels (Figure 6A, B ,andF). Furthermore, the addition of ML385 led to a notable reduction in Nrf2 and HO-1 protein expression and increased the levels of MDA and GSSG (Figure 6C-E). Collectively, these findings confirm that Nrf2 is a key molecular target through which Sal exerts its protective effects against MPA-induced oxidative damage in intestinal epithelial cells.

## 5. Discussion

Mycophenolic acid is a first-line immunosuppressant commonly used in solid organ transplantation, including kidney transplants, and for treating autoimmune diseases (15). However, severe diarrhea caused by MPA leads to dose reduction or discontinuation in approximately 21% to 54.2% of renal transplant patients, which negatively impacts their prognosis (1). Our preliminary clinical observations found that the traditional Chinese medicine WZ significantly reduced the risk of MPA induced diarrhea in kidney transplant patients, and this finding was confirmed in animal experiments (16). Therefore, further exploring the intestinal protective mechanism of the active ingredient in WZ helps improve the prevention and treatment of MPA-induced intestinal injury. Schisanhenol, a major active compound of WZ, has strong antioxidant properties, but its intestinal pharmacological effects remain unknown. This study demonstrated that Sal alleviates MPA-induced damage to the intestinal mechanical barrier.

Apoptosis and loss of TJ proteins are key mechanisms underlying MPA-induced injury (17, 18). Although intestinal epithelial cells renew rapidly, MPA selectively inhibits the de novo purine synthesis pathway needed for enterocyte proliferation, disturbing the balance between cell growth and death (1, 19). Moreover, the TJ structure is critical for resisting pathogen invasion and maintaining barrier homeostasis. ZO-1, located on the cytoplasmic side of the TJ complex, links occludin to the actin cytoskeleton and is essential for barrier integrity (20). Mycophenolic acid significantly downregulates ZO-1 and occludin expression, increasing epithelial permeability. Schisanhenol has been reported to have antiapoptotic effects and to protect neuronal and liver cells from damage (21, 22). Our findings confirm the anti-apoptotic effect of Sal and provide new evidence for the protective effect of Sal on the intestinal barrier. Schisanhenol combined with MPA improves Caco-2 cell viability, increases the expression of the antiapoptotic protein Bcl-2, and restores occludin and ZO-1 expression. These findings suggest that Sal protects intestinal epithelial cells by reducing apoptosis and protecting TJ proteins.

Oxidative stress, resulting from an imbalance between oxidants and antioxidants, leads to intracellular ROS accumulation and plays a central role in intestinal injury. Excess ROS trigger apoptosis and cause phosphorylation and degradation of TJ proteins such as ZO-1, leading to disruption of the TJ complex. It also promotes inflammation, which further damages TJs and increases intestinal permeability (23, 24). Our previous studies confirmed that MPA disrupts the intestinal barrier through ROS accumulation and that the ROS scavenger N-acetylcysteamine (NAC) can alleviate this damage (8). Schisanhenol has been shown to reduce endothelial cell injury by activating AMPK-dependent antioxidant pathways and improve cognitive function in mice by increasing SOD and GSH-Px activity while lowering MDA levels (13, 25). In this study, Sal significantly reduced MPA-induced ROS and MDA accumulation and increased the activities of antioxidant enzymes, such as SOD and CAT, suggesting that Sal protects against oxidative stress.

The transcription factor Nrf2 is a key regulator of cellular antioxidant defenses (26). Under normal conditions, Nrf2 is inactive in the cytoplasm, but oxidative stress triggers its nuclear translocation, where it activates antioxidant enzymes such as HO-1, SOD, and GSH-Px. The Nrf2 maintains the intestinal redox balance and suppresses inflammation and apoptosis. Studies in Nrf2-deficient mice revealed increased inflammation and reduced antioxidant enzyme levels, highlighting its protective role (27). Moreover, Nrf2 activation increases the expression of TJ proteins and prevents their breakdown and apoptosis due to oxidative stress (28, 29). However, the role of MPA or Sal in the regulation of the Nrf2/HO-1 pathway is not yet known. Our results revealed that MPA inhibits the Nrf2/HO-1 pathway and decreases antioxidant activity, whereas Sal abrogates this effect by increasing Nrf2/HO-1 expression and antioxidant enzyme activity. These findings suggest that MPA-induced intestinal barrier damage may involve Nrf2 suppression and that the protective effect of Sal is linked to the activation of the Nrf2/HO-1 pathway. To further confirm the role of Nrf2, we used ML385, a selective Nrf2 inhibitor to block protective effects of Sal (30). ML385 treatment eliminated the antioxidant benefits of Sal, increased ROS, MDA, and GSSG levels, and abrogated the protective effects of Sal on the intestinal barrier. These findings confirm that the Nrf2/HO-1 pathway plays a key role in MPA-induced intestinal injury and that Sal partly protects against this injury by activating this pathway.

Notably, Sal alone was toxic only at 75 μM, but in combination with MPA, toxicity appeared at 50 μM. We hypothesize that MPA-induced intestinal barrier damage may enhance Sal uptake or impair its exocytosis, resulting in increased intracellular Sal concentrations, which could explain its toxicity at 50 μM. Furthermore, MPA-induced cellular alterations may render the cells more sensitive to external stimuli, thereby lowering the threshold for Sal toxicity. These results highlight the need to carefully consider dosing when combining treatments. This study has several limitations: First, the protective effects of Sal need to be validated in vivo; second, the interaction between signaling pathways is complex, and further research is needed to fully elucidate the regulatory mechanisms of Sal.

### 5.1. Conclusions

In conclusion, this study demonstrated for the first time that MPA mediates oxidative stress injury in intestinal epithelial cells by inhibiting the Nrf2/HO-1-regulated antioxidant system, leading to intestinal epithelial cell apoptosis and the downregulation of TJ expression. In contrast, Sal, the main component of *S. chinensis*, alleviated MPA-mediated oxidative stress injury and protected intestinal epithelial cells by increasing Nrf2 expression. The screening of antioxidant components from natural active ingredients may provide new ideas for the prevention and treatment of adverse reactions related to MPA-induced diarrhea.

ijpr-24-1-161994-s001.pdf

## Data Availability

All data included in this study are available upon request by contacting the corresponding author.
