# Supplementary material for: Schisanhenol Alleviates Mycophenolic Acid-Induced Intestinal Epithelial Cell Barrier Damage by Activating the Nrf2/HO-1 Signaling Pathway
Source: Iran J Pharm Res. 2025 Jul 26;24(1):e161994. doi: 10.5812/ijpr-161994 (PMC12523734; doi:10.5812/ijpr-161994)
Supplement: ijpr-24-1-161994-s001.pdf [file ijpr-24-1-161994-s001.pdf]

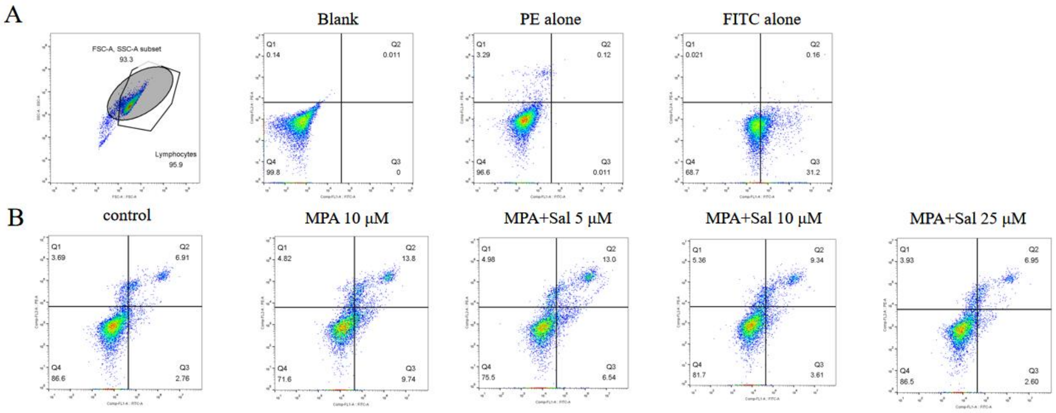

Appendix 1 Caco-2 Cell flow cytometry gating strategy (Apoptosis). (A) Proportion of cells incubated with PE/FITC fluorescent dyes alone or without dyes. (B) Proportion of cells incubated with PE and FITC fluorescent dyes.

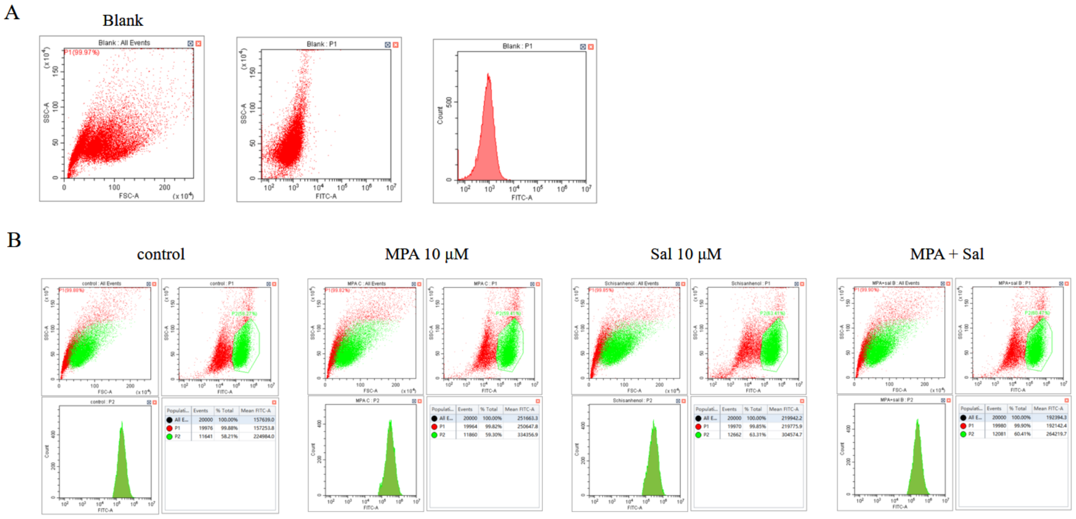

Appendix 2 Caco-2 Cell flow cytometry gating strategy (ROS). (A) Proportion of cells incubated without H_2DCFDA probe. (B) Proportion of cells incubated with H_2DCFDA probe.
